# Effect of remimazolam tosilate on the incidence of hypoxemia in elderly patients undergoing gastrointestinal endoscopy: A bi-center, prospective, randomized controlled study

**DOI:** 10.3389/fphar.2023.1131391

**Published:** 2023-04-18

**Authors:** Fang Liu, Xiaoyan Cheng, Yingjie Wang, Kai Li, Tianliang Peng, Ningning Fang, Kalyan K. Pasunooti, Seungho Jun, Xiaomei Yang, Jianbo Wu

**Affiliations:** ^1^ Department of Anaesthesiology, Qilu Hospital of Shandong University, Jinan, China; ^2^ School of Medicine, Cheeloo College of Medicine, Shandong University, Jinan, China; ^3^ Department of Anaesthesiology, Weifang People’s Hospital (The First Affiliated Hospital of Weifang Medical University), Weifang, China; ^4^ School of Biological Sciences, Nanyang Technological University, Singapore, Singapore; ^5^ Department of Pharmacology and Molecular Sciences, Johns Hopkins School of Medicine, Baltimore, MD, United States; ^6^ Division of Cardiology, Johns Hopkins University Medical Institutions, Baltimore, MD, United States; ^7^ Department of Cardiology, The Key Laboratory of Cardiovascular Remodeling and Function Research, Chinese Ministry of Education, Chinese National Health Commission and Chinese Academy of Medical Sciences, The State and Shandong Province Joint Key Laboratory of Translational Cardiovascular Medicine, Qilu Hospital, Cheeloo College of Medicine, Shandong University, Jinan, China; ^8^ Department of Anaesthesiology and Perioperative Medicine, Qilu Hospital Dezhou Hospital, Shandong University, Dezhou, China

**Keywords:** remimazolam tosilate, propofol, elderly patients, hypoxemia, gastrointestinal endoscopy

## Abstract

**Background:** Remimazolam tosilate is a new ultra-short-acting benzodiazepine sedative medicine. In this study, we evaluated the effect of remimazolam tosilate on the incidence of hypoxemia during sedation in elderly patients undergoing gastrointestinal endoscopy.

**Methods:** Patients in the remimazolam group received an initial dose of 0.1 mg/kg and a bolus dose of 2.5 mg of remimazolam tosilate, whereas patients in the propofol group received an initial dose of 1.5 mg/kg and a bolus dose of 0.5 mg/kg of propofol. Patients received ASA standard monitoring (heart-rate, non-invasive blood pressure, and pulse oxygen saturation) during the entire examination process. The primary outcome was the incidence of moderate hypoxemia (defined as 85%≤ SpO_2_< 90%, >15s) during the gastrointestinal endoscopy. The secondary outcomes included the incidence of mild hypoxemia (defined as SpO_2_ 90%–94%) and severe hypoxemia (defined as SpO_2_< 85%, >15s), the lowest pulse oxygen saturation, airway maneuvers used to correct hypoxemia, patient’s hemodynamic as well as other adverse events.

**Results:** 107 elderly patients (67.6 ± 5.7 years old) in the remimazolam group and 109 elderly patients (67.5 ± 4.9 years old) in the propofol group were analyzed. The incidence of moderate hypoxemia was 2.8% in the remimazolam group and 17.4% in the propofol group (relative risk [RR] = 0.161; 95% confidence interval [CI], 0.049 to 0.528; *p* < 0.001). The frequency of mild hypoxemia was less in the remimazolam group, but not statistically significant (9.3% vs. 14.7%; RR = 0.637; 95% CI, 0.303 to 1.339; *p* = 0.228). There was no significant difference in the incidence of severe hypoxemia between the two groups (4.7% vs. 5.5%; RR = 0.849; 95% CI, 0.267 to 2.698; *p* = 0.781). The median lowest SpO_2_ during the examination was 98% (IQR, 96.0%–99.0%) in patients in the remimazolam group, which was significantly higher than in patients in the propofol group (96%, IQR, 92.0%–99.0%, *p* < 0.001). Patients in the remimazolam group received more drug supplementation during endoscopy than patients in the propofol group (*p* = 0.014). There was a statistically significant difference in the incidence of hypotension between the two groups (2.8% vs. 12.8%; RR = 0.218; 95% CI, 0.065 to 0.738; *p* = 0.006). No significant differences were found in the incidence of adverse events such as nausea and vomiting, dizziness, and prolonged sedation.

**Conclusion:** This study explored the safety of remimazolam compared with propofol during gastrointestinal endoscopy in elderly patients. Despite the increased supplemental doses during sedation, remimazolam improved risk of moderate hypoxemia (i.e., 85%≤ SpO_2_ < 90%) and hypotension in elderly patients.

## 1 Introduction

As life expectancy increases in most parts of the world (World Health Organization, 2021), the incidence of gastrointestinal diseases is increasing in parallel with the aging of the population. Study statistics show that elderly people account for a high proportion of all newly diagnosed gastrointestinal malignancies (colorectal, gastric, esophageal, etc.) (Hall et al., 2005). Gastrointestinal endoscopy is one of the effective methods to diagnose gastrointestinal diseases. Moderate sedation during gastrointestinal endoscopy can reduce patients’ anxiety and discomfort, reduce patients’ memory of the examination process (Igea et al., 2014), and significantly improve endoscopy’s efficacy and safety (Seip et al., 2010; Early et al., 2018). The administration of medications to elderly patients should pay more attention from clinicians. The types of medications used for sedation in the elderly are not different from those used in young patients, but one needs to be aware of the increased sensitivity of this population to medications (Chandrasekhara et al., 2013). Older people are at increased risk for adverse events such as hypoxemia, hypotension, arrhythmias, and esophageal reflux during the examinations (Travis et al., 2012; Shimizu et al., 2021).

Hypoxemia during gastrointestinal endoscopy is a common problem. Due to the non-uniform definition of hypoxemia and the influence of multiple factors such as sedation application, type of endoscopy, and the patient’s condition, the incidence of hypoxemia reported in previous studies varies widely, ranging from 1.4% to 32% (Friedrich-Rust et al., 2014; Dumonceau et al., 2015; Gouda et al., 2017). Severe hypoxemia often requires emergency airway management, such as positive pressure ventilation by mask or tracheal intubation, but this is known to interrupt endoscopic procedures (Beitz et al., 2012). Exploring some effective measures to reduce or prevent the incidence of hypoxemia during gastrointestinal endoscopy has been an enthusiastic issue around the world (Shao et al., 2020).

Over the long term, the acting sedative-hypnotic agent propofol, with its unique lipolytic properties, rapid onset of action, and recovery (Nelson et al., 2001), has brought reasonable satisfaction to users (Cohen et al., 2006). In China, propofol combined with opioids has become the most common form of sedation (Zhou et al., 2021). However, propofol has a narrow therapeutic window, especially in elderly patients, with high risks of common adverse effects such as respiratory depression and hypotension (Shimizu et al., 2021).

Remimazolam, a new ultra-short-acting benzodiazepine derivative, produces sedation by acting on GABA receptors to inhibit the electrical activity of neurons (Keam, 2020). It has a carboxyl ester bond not found in other benzodiazepines, so it can be rapidly metabolized to inactive substances by human tissue enzymes (Sneyd and Rigby-Jones, 2020). Remimazolam was found to be effective for gastrointestinal endoscopy in adult patients, and its sedative effect was not inferior to that of propofol (Chen et al., 2020; 2021). Patients using remimazolam had smoother signs such as blood pressure and heart rate during the examination and showed a higher safety profile in areas such as hypotension, respiratory depression, and injection site pain (Rex et al., 2018; Shi et al., 2022; Xu et al., 2022). However, most studies have been tested in populations with a wide age range and have not explored the drug’s safety in elderly patients. Here, we designed this study to evaluate the safety of remimazolam compared with propofol during gastrointestinal endoscopy in elderly patients, especially concerning the incidence of hypoxemia.

## 2 Materials and methods

### 2.1 Study design

This bi-center, prospective, randomized controlled study was conducted to evaluate the safety of the new benzodiazepine sedative remimazolam tosilate (Hengrui Pharma, China) in elderly patients undergoing painless gastrointestinal endoscopy and to compare it with propofol (Diprivan; AstraZeneca, United Kingdom). Patients in this study were from two tertiary medical centers in Shandong, China, from 1 February 2022, to 30 June 2022.

This study was approved by the Ethics Committee of Qilu Hospital of Shandong University on 22 March 2021 (ethics: KYLL-202011-071-1), and recorded in the Ethics Committee of Weifang People’s Hospital, which participated in this study. It was registered in the Chinese Clinical Trial Registry (registration No: ChiCTR2200056111) on 1 February 2022. Informed consent was obtained from the patient or their representative before each patient performed any procedure.

### 2.2 Participants

Patients aged 60–80 years old, American Society of Anesthesiologists (ASA) physical status I-II, with a body mass index (BMI) of 18–30 kg/m^2^, and who signed informed consent for the study were included in this study. Exclusion criteria included: the presence of diagnosed heart disease (arrhythmia, heart failure, angina, infarction, etc.); the presence of diagnosed lung disease (bronchitis, asthma, COPD, pulmonary herpes, pulmonary embolism, lung cancer, etc.); previous hypotension (systolic blood pressure ≤90 mmHg), bradycardia (heart rate <50 beats/min), or hypoxemia (SpO_2_ <90%); contraindications to gastroscopy (gastric retention, long-term aspirin administration, impaired consciousness, etc.); with underlying diseases requiring oxygen inhalation; definite upper respiratory tract infection; allergic to propofol and other sedative drugs; expected duration of gastrointestinal endoscopy more than 40 min and non-consent to participate in this study.

### 2.3 Randomization and blinding

All eligible participants were randomized by random number table in a 1:1 ratio into the remimazolam group and the propofol group. Remimazolam group given an initial dose of 0.10 mg/kg of remimazolam tosilate and propofol group given an initial dose of 1.5 mg/kg of propofol, respectively. We designed the trial as single-blind due to the different appearance and doses of remimazolam tosilate and propofol. The anesthetists performing the sedation and the data recorders were aware of each patient’s drug treatment assignment throughout the study. All patients were blinded to group assignment.

### 2.4 Study procedures

On the day of the gastrointestinal endoscopy, the patient was given an intravenous infusion channel in the waiting room. After admission to the endoscopy room, the infusion channel was connected, and the monitoring instrument obtained real-time data on ECG, non-invasive blood pressure (every 5 min), and SpO_2_. All patients were placed in a lateral position and inhaled oxygen at an initial value of 5 L/min through a transparent nasal cannula.

All patients were slowly given sufentanil 5ug via intravenous infusion channel, and 2 min later, sedative drugs (single dose 0.10 mg/kg remimazolam tosilate or 1.5 mg/kg propofol) were given according to random group. After full sedation (modified observer alertness/sedation score MOAA/S ≤3), the gastroscopy procedure was started. If sedation was inadequate or if the patient reacted by choking and swallowing during endoscopy placement, a bolus dose (2.5 mg remimazolam for remimazolam group and 0.5 mg/kg propofol for propofol group could be given at least 1 min after the initial dose, with a maximum of two bolus doses allowed. Failure of sedation was recorded if suitable sedation was not obtained after two bolus doses were given. Resuscitation medication (propofol) was given to the patient at the discretion of the anesthesiologist to complete the subsequent gastroscopy procedure. Once the examination has begun, the anesthesiologist may decide to administer a bolus dose of the test drug at least every 1 min (no more than five times cumulatively over 15 min) to maintain the patient at an appropriate depth of sedation (MOAA/S ≤3). At the end of the examination, the anesthesiologist assesses the patient’s consciousness and hemodynamics date, then transfers the patient on a flatbed to the post-anesthesia observation room.

### 2.5 Study outcomes

Patient characteristics (gender, age, height, weight, BMI, ASA classification, etc.) and procedure information (type of procedure) are recorded by asking the patient and reviewing the patient’s medical record. In addition, the examination time (defined as the time between the start of the examination after adequate anesthesia to the end of the examination) was recorded.

The primary outcome was the incidence of moderate hypoxemia during gastrointestinal endoscopy, defined as any occurrence of 85%≤ SpO_2_< 90%, duration >15s. The secondary outcomes included the incidence of mild hypoxemia (defined as SpO_2_ 90%–94%) and severe hypoxemia (defined as SpO_2_ < 85%, duration >15s), the lowest oxygen saturation during the procedure, airway maneuvers used to correct hypoxemia (include increase oxygen flow, lift the jaw, mask positive pressure ventilation, placement of nasopharyngeal ventilation tube, and tracheal intubation ventilation), and patient’s hemodynamic.

We recorded the patient’s hemodynamics at the following time points: before administration of intravenous sufentanil (T0), 5 min after administration of intravenous remimazolam or propofol (T1), 10 min after administration of intravenous remimazolam or propofol (T2), 15 min after administration of intravenous remimazolam or propofol (T3), 20 min after administration of intravenous remimazolam or propofol (T4), end of the gastrointestinal endoscopy (T5).

Record whether the patient has any other adverse events, including but not limited to bradycardia (defined as heart rate below 40 beats/min), hypotension (defined as a 20% or greater decrease in systolic blood pressure from initial values or systolic blood pressure below 80 mmHg), prolonged sedation (defined as MOAA/S score ≤3 after 10 min of sedative discontinuation), postoperative nausea and vomiting, headache, and esophageal reflux. The severity of adverse events was assessed using the Common Terminology Criteria for Adverse Events (CTCAE) version 5.0. (US Department of Health and Human Services, 2017).

The initial dose of sedative medication and the number and dose of supplemental doses given were recorded, and the total amount of sedative medication administered to the patient was calculated.

### 2.6 Sample size calculation

Based on the results of our pre-experiments and relevant literature, this trial used PASS 11.0 software to estimate the sample size using the difference in the incidence of hypoxia (SpO_2_ < 90%, >15s) between the remimazolam group and the propofol group. Setting parameters α = 0.05 and power = 0.8, the expected incidence of hypoxia was 3% (remimazolam group) and 16% (propofol group) (Cai et al., 2017), which was calculated to require 94 patients per group for our study, and taking into account a 10% shedding rate, a minimum sample size of 210 patients (105 each in the remimazolam groups and propofol groups) was finally determined.

### 2.7 Statistical analysis

SPSS 23(IBM, Armonk, NY) software was used for statistical analysis in this trial. Numerical variables involved in the study were analyzed based on normality tests, expressed as mean and standard deviation (SD) or median (Interquartile range, IQR) as appropriate, compared using Student’s t-test or Wilcoxon-Mann-Whitney test. Categorical variables involved were expressed as the number of cases and relative numbers (%). Chi-squared or Fisher’s exact tests were used to compare two groups with categorical characteristics and outcomes. The results were considered statistically significant if *p* < 0.05.

## 3 Results

**FIGURE 1:**
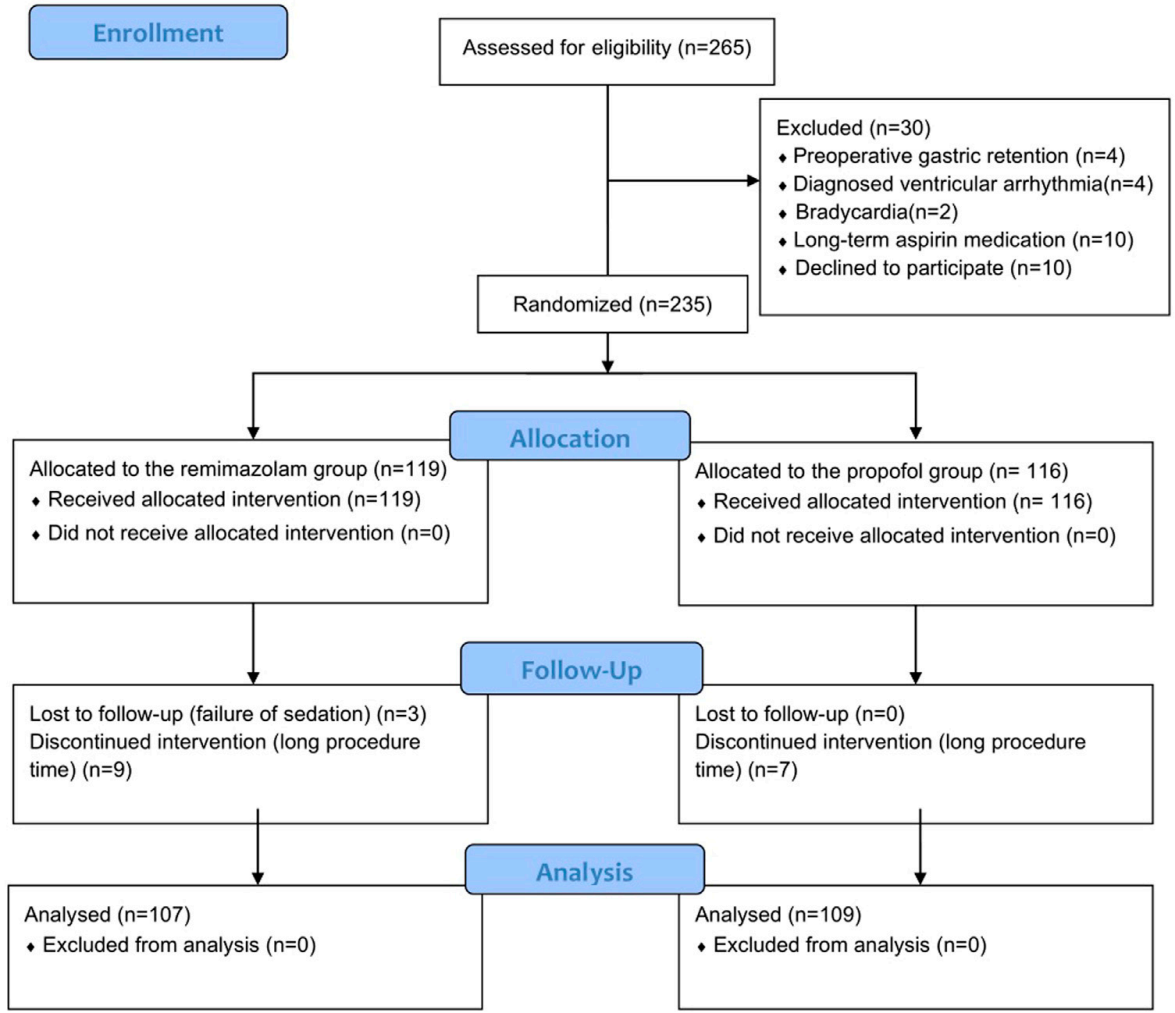
Patient flowchart.

The patient enrollment process for this study shown in Figure 1. We recruited 265 elderly patients, and 30 were excluded (4 had preoperative gastric retention, 4 had diagnosed ventricular arrhythmia, 2 had bradycardia, 10 had long-term aspirin medication, and 10 withdrew informed consent). The remaining 235 patients were randomly divided into two groups. During the procedure, 19 patients were excluded due to failure of sedation induction or excessive examination time. Ultimately, the sample included 107 patients in the remimazolam group and 109 patients in the propofol group.

Patients in both groups for gender, age, height, weight, BMI, ASA score, type of endoscopy, duration of examination, initial heart rate, blood pressure, and oxygen saturation were comparable (Table 1).

**TABLE 1:** Baseline characteristics of the patients and procedure.

Characteristic	Remimazolam group (*n* = 107)	Propofol group (*n* = 109)	*p* value
Age: year, mean (SD)	67.6(5.7)	67.5(4.9)	0.798
Sex: No. (%)			0.898
male	51(47.7)	51(46.8)	
female	56(52.3)	58(53.2)	
Height: m, mean (SD)	1.64(0.08)	1.65(0.08)	0.407
Weight: kg, mean (SD)	63.9(9.9)	65.2(8.4)	0.328
BMI: kg m^-1^, mean (SD)	23.7(3.0)	24.0(2.6)	0.534
ASA: No. (%)			0.784
Grade I	11(10.3)	10(9.2)	
Grade II	96(89.7)	99(90.8)	
Initial heart rate: times/min, mean (SD)	78(10.5)	76(8.9)	0.257
Initial SpO_2_, %, median (IQR)	99.0(98.0–100.0)	98.0(98.0–100.0)	0.800
Initial systolic pressure: mmHg, mean (SD)	148(22.5)	144(15.7)	0.252
Initial diastolic pressure: mmHg, mean (SD)	83(14.5)	82(10.4)	0.746
Procedure category: No. (%)			0.652
Gastroscopy	40(37.4)	45(41.3)	
Colonoscopy	16(15.0)	12(11.0)	
Gastrointestinal endoscopy	51(47.7)	52(47.7)	
Duration of procedure: min, mean (SD)			
Gastroscopy	9.6(5.2)	8.3(3.1)	0.169
Colonoscopy	21.6(7.0)	17.2(4.9)	0.075
Gastrointestinal endoscopy	24.7(7.5)	24.5(7.5)	0.936

ASA, american society of anesthesiologists physical status; BMI, body mass index; SD, standard deviation; IQR, interquartile range.

### 3.1 Study outcome

**FIGURE 2:**
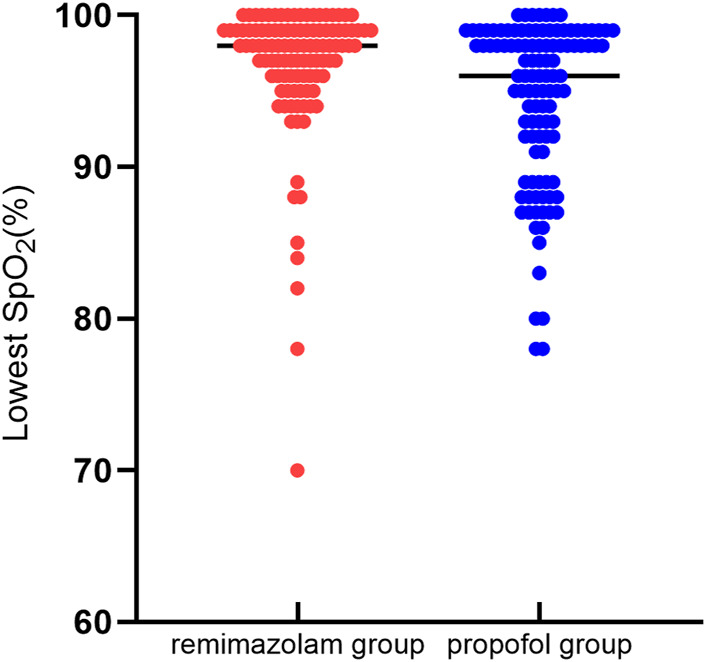
Lowest SpO_2_ comparison. Distribution of the lowest intraoperative oxygen saturation values in 107 patients in the remimazolam group and 109 patients in the propofol group. Analysis by the Wilcoxon-Mann-Whitney test revealed a statistical difference between the two groups (*p* < 0.05).

The incidence of moderate hypoxemia was 2.8% in the remimazolam group and 17.4% in the propofol group, with a statistically significant difference between the groups (relative risk [RR] = 0.161; 95% confidence interval [CI], 0.049 to 0.528; *p* < 0.001). The frequency of mild hypoxemia was less in the remimazolam compared to the propofol group but without statistically significant (9.3% vs. 14.7%; RR = 0.637; 95% CI, 0.303 to 1.339; *p* = 0.228). Severe hypoxemia incidence did not differ significantly between the two groups (4.7% vs. 5.5%; RR = 0.849; 95% CI, 0.267 to 2.698; *p* = 0.781). The median lowest SpO_2_ was 98% (interquartile range [IQR], 96.0%–99.0%) in the remimazolam group and 96% (IQR, 91.5%–99%) in the propofol group, which was statistically significantly different (*p* < 0.001). Hypoxemia in both groups could be corrected by increasing oxygen flow or lifting the jaw, and one case in the remimazolam group required interruption of endoscopy to correct severe hypoxemia. (Table 2; Figure 2).

**TABLE 2:** Comparison of hypoxemia and interventions.

	Remimazolam group (*n* = 107)	Propofol group (*n* = 109)	RR (95% CI)	*p* value
moderate hypoxemia, n (%)	3(2.8)	19(17.4)	0.161(0.049–0.528)	<0.001
severe hypoxemia, n (%)	5(4.7)	6(5.5)	0.849(0.267–2.698)	0.781
mild hypoxemia, n (%)	10(9.3)	16(14.7)	0.637(0.303–1.339)	0.228
Lowest SpO_2_, %, median (IQR)	98.0(96.0–99.0)	96.0(91.5–99.0)	—	<0.001
Emergency airway management, n (%)			—	0.005
Increase oxygen flow	0(0.0)	3(2.8)		
Lift the jaw	7(6.5)	22(20.2)		
Mask positive pressure ventilation	1(0.9)	0(0.0)		
Nasopharyngeal ventilation tube	0(0.0)	0(0.0)		
Tracheal intubation or laryngeal mask	0(0.0)	0(0.0)		

Moderate hypoxemia: 85%≤ SpO_2_ <90%, >15s; severe hypoxemia: SpO_2_<85%, >15s; mild hypoxemia: SpO_2_ 90%–94%; RR, relative risk; CI, confidence interval; IQR, Interquartile Range.

### 3.2 Hemodynamic

**FIGURE 3:**
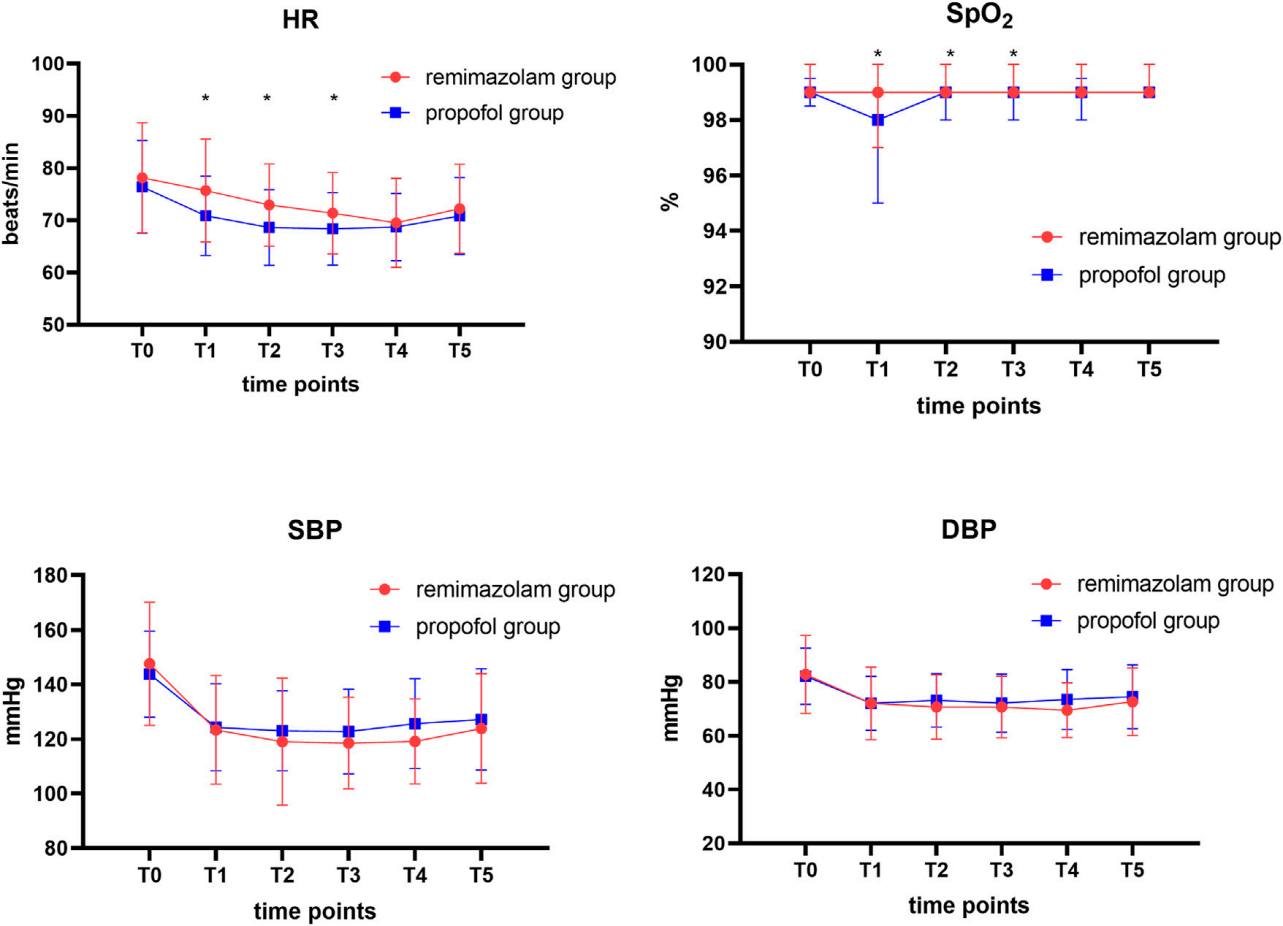
Comparison of hemodynamic profiles and oxygen saturation levels in two groups at different time points. Data are expressed as mean ± SD or median [IQR]. HR, heart rate; SBP, systolic blood pressure; DBP, diastolic blood pressure. T0, before administration of intravenous sufentanil; T1, 5 min after administration of intravenous remimazolam or propofol; T2, 10 min after administration of intravenous remimazolam or propofol; T3, 15 min after administration of intravenous remimazolam or propofol; T4, 20 min after administration of intravenous remimazolam or propofol; T5, end of the gastrointestinal endoscopy. The Wilcoxon-Mann-Whitney test was used to compare the differences in oxygen saturation between the two groups of patients. *p < 0.05 considered a statistical difference between the two groups.

We recorded the hemodynamic parameters of the patients at each time point during the endoscopy. Trends in blood pressure changes at each time point during the endoscopy procedure were similar and not statistically significantly different. However, there were statistically significant differences (*p* < 0.05) in pulse oxygen saturation and heart rate data between the two groups of patients at 5, 10, and 15 min after sedation drug administration (T1, T2, T3) (Figure 3).

### 3.3 Medicine dosage

Table 3 showing the dosage of sedative medicine in both groups. Among the 109 elderly patients in the propofol group, most patients achieved the target sedation level (MOAA/S ≤3) at the scheduled initial dose, and only two patients received a bolus dose before starting endoscopy. In contrast, the initial dose of 0.1 mg/kg in the remimazolam group failed to meet sedation requirements in some patients, with 55 patients receiving one bolus dose and 13 patients receiving two bolus doses before beginning endoscopy procedures. Patients in the remimazolam group received more drug supplements during the endoscopy (21/24/25/21/6/10 vs. 42/20/26/14/5/2 patients requiring 0, 1, 2, 3, 4 or> 4 bolus doses, *p* = 0.014). The two group’s total doses of sedative drugs were 14.1 ± 4.82 mg vs. 140.0 ± 47.15 mg, respectively.

**TABLE 3:** Comparison of sedative drug dosages.

	Remimazolam group (*n* = 107)	Propofol group (*n* = 109)	*p* value
Successful sedation in initial dosage: No. (%)	39(36.4)	107(98.2)	<0.001
Sedation induction dosage: mg, mean (SD)	8.3(1.9)	98.3(13.1)	<0.001
Additional: No. (%)			0.014
0	21(19.6)	42(38.5)	
1	24(22.4)	20(18.3)	
2	25(23.4)	26(23.9)	
3	21(19.6)	14(12.8)	
4	6(5.6)	5(4.6)	
>4	10(9.3)	2(1.8)	
Total dosage: mg, mean (SD)	14.1(4.8)	140.0(47.2)	<0.001

Induction dosage: initial dosage + up to two bolus doses; Total dosage: induction dose + all additional bolus doses; SD, standard deviation.

### 3.4 Other adverse events

Adverse events occurred in 30 patients (28%) in the remimazolam group and 38 patients (34.9%) in the propofol group, with no significant differences found (RR = 0.804; 95% CI, 0.540 to 1.197; *p* = 0.280). The incidence of intraoperative hypotension was significantly lower in patients in the remimazolam group (2.8% vs. 12.8%; RR = 0.218; 95% CI, 0.065 to 0.738; *p* = 0.006). In addition, nausea and vomiting were the most common postoperative adverse events in both groups, accounting for 10.3% vs. 6.4% of the total incidence, respectively, but there was no significant statistical difference (RR = 1.601; 95% CI, 0.645 to 3.974; *p* = 0.305). According to the Common Terminology Criteria for Adverse Events (CTCAE) v5.0, all adverse events in the study were mild or moderate, and no serious adverse events occurred. (Table 4).

**TABLE 4:** Incidence of adverse events.

	Remimazolam group (n = 107)	Propofol group (n = 109)	RR (95% CI)	*p*-value
Any adverse events, n (%)	30 (28.0)	38 (34.9)	0.804 (0.540–1.197)	0.280
Nausea and vomiting, n (%)	11 (10.3)	7 (6.4)	1.601 (0.645–3.974)	0.305
Dizziness/headache, n (%)	4 (3.7)	4 (3.7)	1.019 (0.261–3.969)	0.979
Prolonged sedation, n (%)	7 (6.5)	7 (6.4)	1.019 (0.370–2.806)	0.971
Hypotension, n (%)	3 (2.8)	14 (12.8)	0.218 (0.065–0.738)	0.006
Bradycardia, n (%)	5 (4.7)	6 (5.5)	0.849 (0.267–2.698)	0.781

Prolonged sedation: MOAA/S score ≤3 after 10 min of sedative discontinuation; Hypotension: a 20% or greater decrease in systolic blood pressure from initial values or systolic blood pressure below 80 mmHg; Bradycardia: heart rate below 40 beats/min; RR, relative risk; CI, confidence interval.

## 4 Discussion

This randomized controlled study involving 216 elderly patients evaluated the safety of remimazolam in elderly patients undergoing gastrointestinal endoscopy. Compared with propofol, elderly patients sedated using remimazolam tosilate had a lower risk of hypoxemia (i.e., SpO_2_<90%) and hypotension during gastrointestinal endoscopy, despite the increased number of peri-procedural bolus doses. Furthermore, Remimazolam did not increase the risk of other adverse effects such as PONV, dizziness/headache, prolonged sedation, and bradycardia in elderly patients.

Many factors cause hypoxemia during gastrointestinal endoscopy. When the endoscope lens is placed in the esophagus, pharyngeal obstruction or tracheal compression may occur (Rimmer et al., 1989). Age is independently associated with hypoxemia during gastroscopy (Travis et al., 2012); with increasing age, arterial partial pressure of oxygen decreases gradually due to a mismatch between ventilation and perfusion. In addition, the elasticity of the lungs decreases with age, and the risk of airway collapse is higher in the elderly (Boss and Seegmiller, 1981). Previous studies have demonstrated that sedation significantly increases the incidence of desaturation and hypoxia during gastrointestinal l endoscopy (Reed et al., 1993; Wang et al., 2000). The application of sedative drugs causes central nervous system depression, producing a higher incidence of respiratory depression and apnea, which undoubtedly exacerbates the risk of hypoxia during examination in elderly patients. Severe hypoxemia leads to increased anaerobic metabolism and changes in the circulatory system (e.g., arrhythmias, myocardial ischemia), and elderly patients with cardiopulmonary disease are at higher risk of hypoxic heart injury (van Schaik et al., 2021).

Effective and safe sedative drugs for gastrointestinal endoscopy are still being explored. Midazolam is a classical benzodiazepine sedative-hypnotic with superior amnesic effects. However, compared with propofol, midazolam induces a longer onset of action and recovery time, which affects the efficiency of gastroscopy room transit (Kim et al., 2021). Etomidate is a common anesthesia-inducing drug, which has mild cardiovascular and respiratory effects and causes fewer cardiopulmonary adverse events. Nevertheless, the high incidence of events such as muscle tremor and postoperative nausea and vomiting affects its use in sedation (Meng et al., 2016). Although propofol is a short-acting anesthetic, it can induce deeper sedation and has no specific reversal agent; patient monitoring should be enhanced during sedation (Conigliaro et al., 2017). In this study, we selected propofol and remimazolam to compare sedation safety in the elderly population. The results showed that intravenous sedation with remimazolam significantly reduced the incidence of hypoxemia during gastrointestinal endoscopy examination in elderly patients.

Remimazolam, a new benzodiazepine derivative, was first reported in 2007. Like other classical benzodiazepines, it binds with high affinity to the gamma-aminobutyric acid receptors in the human central nervous system and can also be antagonized by flumazenil, making it an effective sedative (Kilpatrick et al., 2007). Studie have found that the use of a larger dose of remimazolam may affect postoperative cognitive function in elderly patients (Tan et al., 2022), so the initial dose of remimazolam in our study was determined to be 0.1 mg/kg. In a study conducted by Guo et al., a comparison was made between remimazolam and propofol in elderly patients undergoing gastrointestinal endoscopy. The study found that the use of remimazolam significantly reduced the incidence of respiratory depression and hemodynamic events, thereby confirming its safety as a sedative (Guo et al., 2022). Additionally, despite the difference in initial dose of remimazolam between Guo’s study (i.e., 0.15 mg/kg) and ours (i.e., 0.1 mg/kg), both studies reported a higher number of peri-procedural remimazolam supplemental doses. This observation is consistent with the unique pharmacokinetic properties of remimazolam, which is not dependent on hepatic and renal metabolism and has a short half-life. The accumulation of drugs brings about changes in the depth of sedation and adverse effects. Although patients in the remimazolam group received more intraoperative supplementation to maintain the appropriate depth of sedation, the incidence of hypoxemia remained low compared with propofol. The incidence of adverse effects was highly correlated with the depth of sedation. Several previous clinical studies have confirmed the difference in sedation levels between remimazolam and common drugs such as propofol by recording the curve of the patient’s MOAA/S score over time during endoscopy (Borkett et al., 2015; Pambianco et al., 2016; Chen et al., 2021). The depth of sedation induced by remimazolam is shallow compared to that of propofol. The deeper the depth of sedation, the more pronounced the inhibition of the circulatory and respiratory systems, which explains the high rate of cardiopulmonary adverse events caused by propofol. During the study, the hypoxia of patients in the two groups was also relatively controllable, which could be relieved after adjusting the oxygen flow or lifting the jaw. There were no serious cases requiring temporary withdrawal from the endoscope and emergency mask positive pressure ventilation. Because of the low incidence of hypoxia in the remimazolam group, it means that the number of patients in this group who need airway intervention is small. Patients can receive gastrointestinal endoscopy smoothly under sedation, which reduces the additional stimulation of airway intervention for patients. In conclusion, the application of remimazolam can achieve the depth of sedation required for endoscopy, while this level of sedation is relatively safe, and sedation-related hypoxia events occur less frequently, which is undoubtedly beneficial in the elderly.

In our study, patient’s hemodynamic data collected during endoscopy were analyzed, it was found that the fluctuation of heart rate and blood pressure was less in the remimazolam group than in the propofol group, and the risk of hypotension was lower in the remimazolam group compared to propofol group (2.8% vs.12.8%). This seems to indicate that, compared with propofol, remimazolam has less severe circulatory effects and is more beneficial to the safety of patients during sedation. Retroactively, we think differences in sedation depth may cause it. The pharmacokinetic changes associated with aging complicate the situation in the elderly. The prolonged half-life of lipophilic drugs such as propofol, coupled with the decline of liver and kidney function, may lead to prolonged recovery time after sedation and lead to some adverse events (Nonaka et al., 2015). Remimazolam is a new medication and in order to be widely used in clinical practice, its safety and pharmacokinetic properties need to be evaluated in different populations and at different doses. The study by Antonik et al. found that intravenous infusion of remimazolam was well tolerated in healthy subjects, with no significant difference in the incidence of adverse events compared to midazolam (Antonik et al., 2012). Among elderly patients undergoing gastroscopy, no significant differences were also seen in the incidence of nausea and vomiting, dizziness, and headache with remimazolam compared with propofol (Guo et al., 2022). In this study, no serious adverse effects occurred after sedation in either group of patients, and all types of adverse effects were mild. The most common adverse effects during the postoperative observation period were nausea and vomiting. Although the frequency of this event was higher in the remimazolam group, it was not significantly different from that in the propofol group. We speculate it may be caused by the accumulation of additional drugs in the body. Considering the economic reasons of the patients, we did not collect and analyze the relevant indicators in the patient’s blood.

Our study has the following limitations: First, this study was a single-blind trial and the anesthesiologist performing the sedation was not blinded to the patient grouping, which may have introduced some bias into the trial results. Second, the study lacks some indicators, such as endoscopists, anesthesiologists, and patient’s satisfaction with sedation procedures. Adverse events closely related to sedative drugs such as agitation and delirium during the recovery period were also not observed in the elderly patients in this study. Third, this study only aimed at the application of remimazolam in ordinary gastrointestinal endoscopy, and the examination procedure is short. Whether this drug can be used as safe and effectively applied in some relatively time-consuming gastrointestinal endoscopies, or treatment procedures needs further research to analyze and explore.

## 5 Conclusion

In conclusion, for patients aged 60-80, compared with propofol, the application of remimazolam for sedation during gastrointestinal endoscopy significantly reduced the incidence of hypoxemia. Remimazolam increases the safety of gastrointestinal endoscopy in elderly patients and maybe a new option for sedation medication.

## Data Availability

The raw data supporting the conclusion of this article will be made available by the authors, without undue reservation.
